# The impact of capsular repair on the risk for dislocation after revision total hip arthroplasty – a retrospective cohort-study of 259 cases

**DOI:** 10.1186/s12891-018-2242-0

**Published:** 2018-08-31

**Authors:** Julia Jurkutat, Dirk Zajonz, Gerald Sommer, Stefan Schleifenbaum, Robert Möbius, Ronny Grunert, Niels Hammer, Torsten Prietzel

**Affiliations:** 1 Department of Orthopaedics, Trauma and Plastic Surgery, University Hospital Leipzig, Liebigstrasse 20, D-04103 Leipzig, Germany; 2 Department of Orthopaedics and Trauma Surgery, HELIOS Clinic Blankenhain, Wirthstrasse 5, D-99444 Blankenhain, Germany; 3 ZESBO – Zentrum zur Erforschung der Stütz- und BewegungsOrgane, Semmelweisstrasse 14, D-04103, Leipzig, Germany; 4 Department of Anatomy, University of Otago, Lindo Ferguson Building, 270 Great King St, Dunedin, 9016 New Zealand; 5 Department of Anatomy, University of Leipzig, Semmelweisstraße 14, D-04103 Leipzig, Germany; 6 Fraunhofer Institute for Machine Tools and Forming Technology, 44, Nöthnitzer Straße, D-01187 Dresden, Germany

**Keywords:** Capsular repair, Dislocation, Revision, THA

## Abstract

**Background:**

Dislocation following total hip arthroplasty has to date not been resolved satisfactorily. Previous work has shown that using a less-invasive adaption of Bauer’s lateral transgluteal approach with capsular repair significantly reduces dislocation rates in primary total hip arthroplasty. The aim of this retrospective cohort study was to assess whether this approach also helps to reduce the dislocation rate in revision total hip arthroplasty.

**Methods:**

We analyzed revision total hip arthroplasty cases performed between 10/2005 and 12/2013 in our department, classifying capsular repair cases as study group and capsular resection cases as control group. The WOMAC score, the dislocations and the revisions were observed.

**Results:**

A total of 259 cases were included, 100 in the study group and 159 in the control group. In the 12-month follow-up, dislocation rates were significantly lower in the study group (3%, *n* = 3) compared to the control group (21.4%, *n* = 34; *p* = 0.001). Overall follow-up periods were 49 and 79 months, revision frequencies were 10 and 29%, pain improvements were 5.5 compared to 4.4 and the WOMAC global scores averaged 2.0 ± 2.1 and 2.9 ± 2.6 for the study group and the control group, respectively.

**Conclusion:**

The modified, less-invasive, lateral transgluteal approach with capsular repair was accompanied by an 86% reduction in dislocation rates when compared to the conventional technique with capsular resection via the anterolateral Watson-Jones-approach. Capsular repair is possible in about 60% of the revision total hip arthroplasty cases, may be considered as beneficial to avoid dislocation and can therefore be recommended.

## Background

Total hip arthroplasty (THA) is among the most frequently performed surgical procedures in the industrial world and was titled the operation of the twentieth century [1], providing a leap in patient satisfaction and quality of life in relation to hip-arthritis.

Nevertheless, THA dislocation remains a feared incident, representing the second leading complication and cause of revision surgery following aseptic THA loosening [2–5]. It even predominates in the early postoperative period – a devastating experience for both the affected patient and the responsible surgeon.

The risk of THA dislocation is usually reported to be between 7.5 and 14.4% after Revision THA (R-THA) - two to three times as high as after primary THA [6–9]. In reality, the dislocation rate is likely to be even higher, as not all dislocations can be recorded [10]. Thus, THA dislocation remains an ongoing problem that has yet to be solved.

Avoidance of capsular resection was already recommended in the early days of THA [11, 12]. Nevertheless, quite a few surgeons routinely excised it during R-THA. Current literature suggests that capsular repair [13, 14] and the use of larger prosthetic heads [15, 16] decrease the risk of THA dislocation.

To date, few studies exist on the benefits or disadvantages of capsular repair in R-THA [7, 17, 18] via the posterolateral approach.

Aiming at reduced dislocation rates, a less-invasive adaption of Bauer’s lateral transgluteal approach was developed and deployed. In addition to the typical partial detachment and reconstruction of the ventral part of the iliotrochanteric muscles this procedure was focused on capsular repair, preserving the acetabular origin of the hip joint capsule. This modified technique has been shown to reduce dislocation rates by 88% in primary THA [13]. Since 10/2005 the technique has also been used in R-THA.

The aim of this study was to retrospectively analyze if this modified capsule-preserving technique could likewise reduce the dislocation rate after R-THA. We also wanted to determine whether this technique had adverse effects on the Western Ontario and McMaster Universities Osteoarthritis Index (WOMAC) score and pain scale change, clinical outcomes or subsequent revisions.

## Methods

For decades, the conventional R-THA surgery with capsular resection has been successfully carried out in our department. In 2002, a modified, less invasive surgical technique with capsular repair was additionally introduced, based on the lateral transgluteal (Bauer) approach. Therefore, all R-THA cases treated in our Department in the timeframe between 10/2005 and 12/2013 were identified via German Procedure Classification code 5–821 (‘*revision, exchange and removal of hip prosthesis*’, German modification of International Classification of Procedures in Medicine), included and retrospectively analyzed after approval of the study by the ethics committee of the University of Leipzig (044/14032016).

R-THA was defined as a surgical procedure involving an exchange of the acetabular component and/or liner, femoral stem and/or modular head, or any combination thereof.

**Fig. 1:**
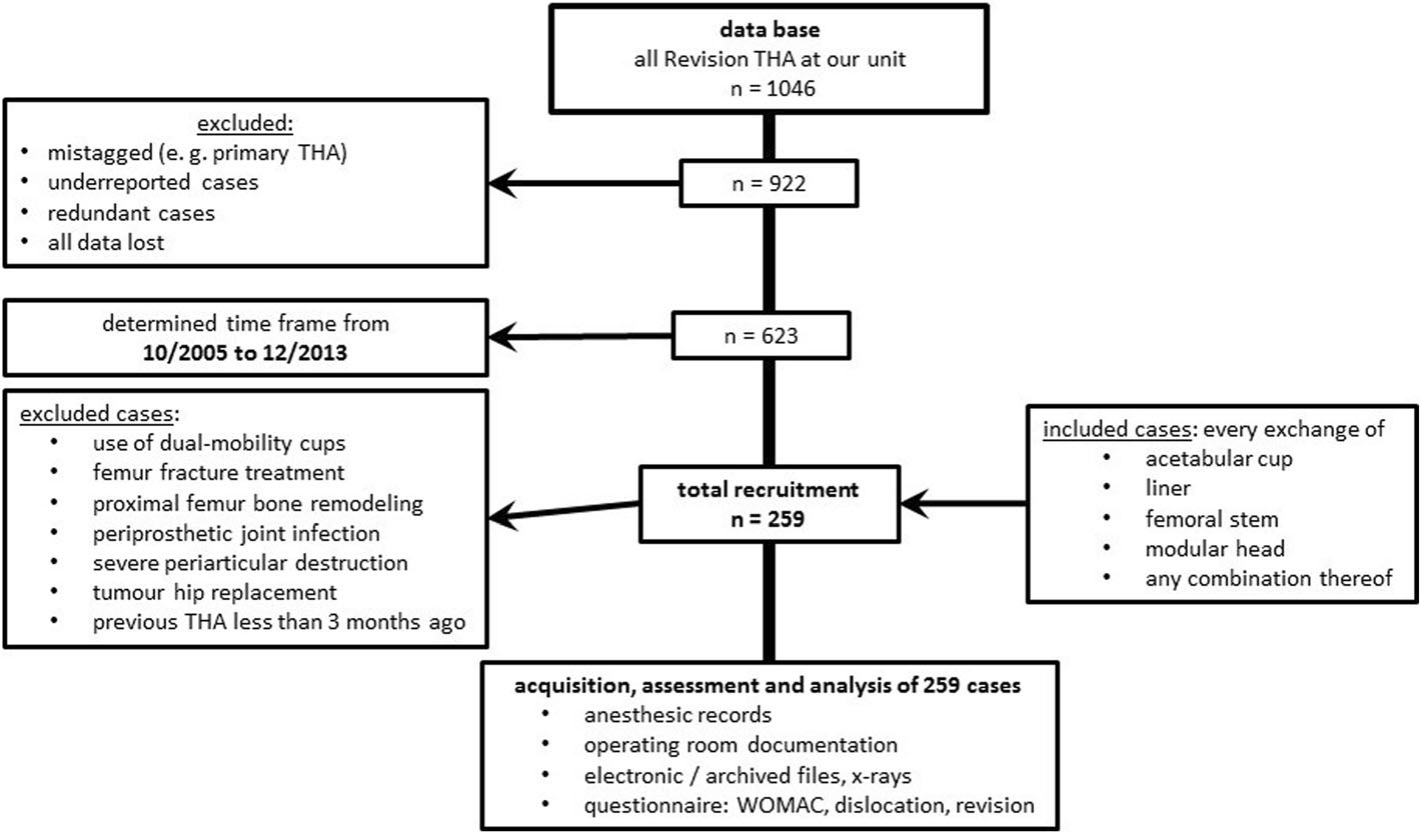
Flow chart: Selection of cases

Patients with dual-mobility cups, periprosthetic femur fracture, proximal femur replacement, periprosthetic joint infection, severe periarticular destruction, tumour disease or recent THA procedures (in the last 3 months) were excluded. The last exclusion criterion was used because of the assumption that the scarred regeneration of a neo-capsule takes at least 3 months [19]. Capsular repair cases were gathered in the study group (SG), capsular resection cases in the control group (CG) (see flow chart Fig. 1). One of the surgeons always aimed for a capsular repair, with the final decision taken during the operation, depending on whether it was feasible. The other surgeons involved routinely resected the capsule. Whether a capsular repair was sought or not was dependent on the scheduled surgeon and thus random. However, in terms of feasibility, selection bias is not completely excluded because the non-reparability of the capsule was most often caused by severe periarticular destruction.

Further patient-related data were taken from the anaesthesia records. A written consent declaration and a questionnaire were both sent to every patient. The latter was focused on the acquisition of the WOMAC score [20], the pre-operative and postoperative elevation of pain scale (retrospectively collected at the time of survey), the detection of dislocation incidents and further revision surgery. All non-responders were additionally contacted via telephone. In cases where the patients themselves were unable to give information (e.g. deceased patients), close relatives (spouse, partner, siblings, children) were asked to answer the key questions of dislocation and revision surgeries. Moreover, all available documentation and clinical imaging data were reviewed for potential dislocation incidents and revision surgery. A dislocation was considered if confirmed by X-ray or documented by a medical specialist. The date of the first dislocation was recorded to calculate dislocation rates in the 6-months and the 12-months follow-up. The appropriate medical case files were requested in cases where a closed reduction or revision surgery was performed elsewhere. The target variables were postoperative dislocation rates in the 12-months follow-up and total revision rates. If a revision occurred within 6 months after a dislocation of the same hip joint, it was rated as ‘re-revision due to dislocation’. Furthermore, the cutting-to-suture time, the postoperative hospital stay time as well as a pain scale and WOMAC indices were determined as secondary parameters.

In the timeframe between 10/2005 and 12/2013 623 R-THA-cases were treated in our department. Two hundred fifty-nine of this met the inclusion criteria and did not fulfil the exclusion criteria of this study. One hundred R-THA-cases were treated in the less-invasive capsule preserving technique via Bauer’s lateral approach [21] and thus included in the SG. One hundred fifty-nine R-THA-cases were treated in the conventional technique with capsular resection via the anterolateral Watson-Jones-approach [22] and thus included in the CG.

### Less-invasive surgical technique in R-THA including capsular repair

**Fig. 2:**
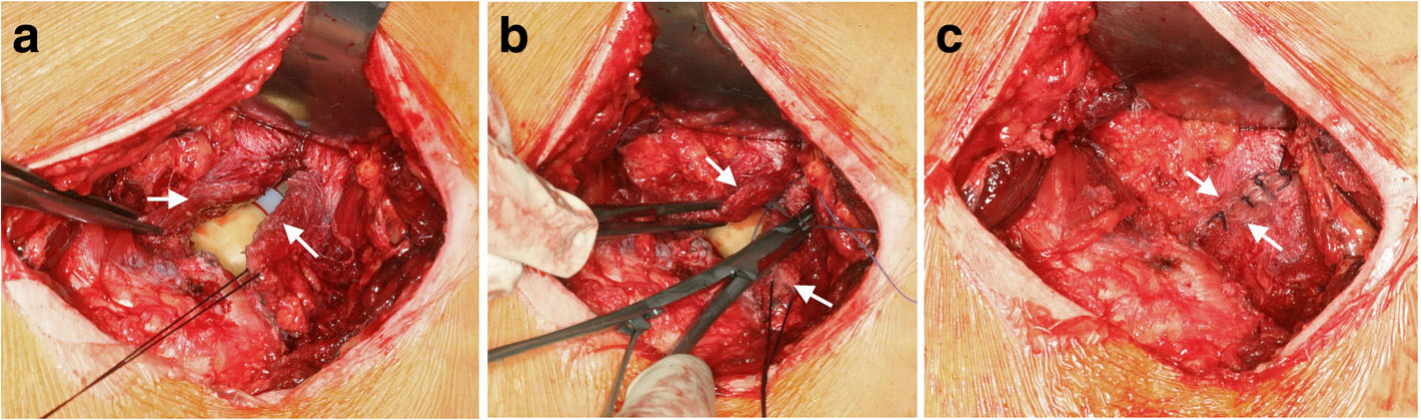
Surgical technique of capsular repair (left hip joint): **a** Both capsular flaps (arrows) are neared by, **b** The ventral longitudinal incision is closed by suture, **c** Capsular repair is finished

The modified technique was based on Bauer’s lateral transgluteal approach [21], which is characterised by a lateral longitudinal skin incision and the detachment of the ventral part of the muscles, which attaches or originates ventrally to the greater trochanter. Connective tissues in the plane of the hip joint capsule are incised in an inverted T-shape manner, preserving its acetabular origin completely [13]. In most cases the capsule was additionally incised cranially longitudinally, forming a U-flap of its ventrocranial section. In a few cases the removal of the internal layer of the capsule was necessary, while the outer layer was preserved. After the implantation and reduction of the prosthesis, both longitudinal capsular incisions (ventral and cranial) were closed by sutures (Fig. 2), while the transverse incision near the femoral neck base remained unclosed and served for insertion of a Redon drain [13]. After reconstruction of the muscles, the fascia and the subcutaneous layer the skin was routinely closed by a resorbable intracutaneous suture, so that no removal of suture material was necessary.

### Conventional surgical technique in R-THA including capsular resection

The technique was based on the anterolateral approach (Watson-Jones-approach [22]).

The skin incision is slightly curved from distal-lateral to proximal-ventral. After the incision of the Tractus iliotibialis, the Musculus gluteus medius and minimus were incised near their attachment to the great trochanter and transverse to the direction of the muscle fibres. The connective tissues in the plane of the hip joint capsule were excised as completely as possible. Following the implantation and reduction of the prosthesis, a Redon drain was inserted and the muscles, the fascia and the subcutaneous layer were reconstructed. Finally, the skin was closed with clips that were removed after 10 days.

### Statistics

Statistical analysis was carried out using EXCEL 2007 (Microsoft Corporation, Redmond, WA, USA) and SPSS STATISTICS version 23 (2015, IBM - International Business Machines Corporation, Armonk, NY, USA). The SG and the CG were compared with regards to epidemiological, implant-associated and surgery-specific data. Statistical evaluations were performed using *independent Student’s t-test, chi-square-test and Cox regression analysis*. *p*-values of 0.05 or less were considered statistically significant, *p*-values of 0.01 or less were considered highly statistically significant.

## Results

### Patient cohort

Two hundred fifty-nine cases were included: 100 were identified retrospectively as belonging to the SG and 159 as belonging to the CG. The collected data and results comparing both groups are shown in Tables 1, 2, 3 and 4. One patient of CG (0.6%) died 11 months after surgery. A total of 45 patients (17%) passed away in the overall follow-up. The notes of the deceased were examined. In total 16 other patients were lost in the follow-up.

**Table 1:** Epidemiologic Data

		Study group	Control group	*p*-value
Number of included cases		100	159	
Number of answered questionnaires		86 (86%)	112 (70%)	
Number of deceased patients		10 (10%)	35 (22%)	
Mean follow-up time		49 months min: 16 max: 90	79 months min: 23 max: 120	
Mean age at surgery		69.6 years min: 41 max: 87	68.5 years min: 23 max: 92	0.386
Mean body mass index (BMI)		28.3 min: 15.9 max: 42.5	28.6 min: 18.5 max: 63.8	0.726
ASA-classification	mean	2.4	2.5	0.011
	1	4%	Ø	
	2	56%	48%	
	3	40%	52%	
Gender male (♂) / female (♂)		♂ 41 (41%) ♀ 59 (59%)	♂ 57 (36%) ♀ 102 (64%)	
Side of procedure right (R) / left (L)		R 52 (52%) L 48 (48%)	R 84 (53%) L 75 (47%)	0.899
Number of previous revisions				0.015
none		93%	78%	
1		6%	17%	
≥ 2		1%	5%

**Table 2:** Surgery- and Implant-Related Data

	Study group	Control group	*p*-value
Number of involved surgeons	1	7	
Mean experience of the surgeon in the field of orthopaedics at the time of surgery	17.2 ± 2.0 years	21.8 ± 7.7 years	
Mean acetabular cup size	56.6 mm min: 48 mm max: 68 mm	57.7 mm min: 48 mm max: 70 mm	0.085
Mean femoral head size	32.9 mm	32.7 mm	0.580
28 mm	14%	7%	
32 mm	50%	72%	
36 mm	36%	19%	
40 mm	Ø	Ø	
44 mm	Ø	1%	
48 mm	Ø	1%	
Mean femoral head size of dislocated cases	32.0 mm (*n* = 3)	33.0 mm (*n* = 34)	
Fixation:			0.001
Uncemented	19%	48.5%	
Hybrid (cemented stem)	20%	13%	
Inversed hybrid (cemented cup)	4%	2%	
Cemented	35%	15%	
No fixation (exchange of head or liner)	22%	21.5%	
Exchange of following components:			0.011
Femoral head and / or acetabular liner	22%	21%	
Acetabular cup	12%	25%	
Femoral stem	24%	14%	
Both cup and stem replacement	42%	40%	
Mean cutting-to-suture time	157 min min: 75 min max: 293 min	172 min min: 54 min max: 439 min	0.042
Mean period of post-operative hospitalization	9 d min: 5 d max: 19 d	17 d min: 6 d max: 83 d	0.001

**Table 3:** Results in the 12-months follow-up

	Study group	Control group	*p*-value
Dislocation after R-THA 12-months follow-up	**3% (*n*** **= 3)**	**21.5% (*n*** **= 34)**	0.001
Dislocation after R-THA 6 months follow-up	**3% (*n*** **= 3)**	**19.5% (*n*** **= 31)**	0.001
Number of dislocations 12 months follow-up			
1	2%	8%	
2	1%	5%	
≥ 3	Ø	8%

**Table 4:** Results in the overall follow-up

	Study group	Control group	*p*-value
Mean overall follow-up time	49 months min: 16 max: 90	79 months min: 23 max: 120	
Cases with re-revision	10% (*n* = 10)	29% (*n*= 46)	
Revision due to dislocation	Ø (*n*= 0)	11% (*n*= 17)	
Time to re-revision			
Within 3 months	1%	16%	
Within 3 to 6 months	2%	2%	
More than 6 months	7%	11%	
Rate of revision surgeries			
1	9%	23%	
2	ø	4%	
≥ 3	1%	2%	
Average pre-operative pain score	7.38	7.22	
Average post-operative pain score	1.91	2.83	
Difference of post−/pre-op. pain score	- 5.48	- 4.41	
Mean WOMAC			
Pain	1.47	2.32	
Stiffness	1.88	2.69	
Physical function	2.66	3.76	
Total score	2.00	2.92	

Thus, follow-up rate was 198 out of 259 (76%). The mean overall follow-up times were 49 months and 79 months (*p* < 0.001) for the SG and CG, respectively, with at least 12 months in all cases except one.

With respect to the epidemiological data there were no significant differences except for the co-existing morbidities classified according to the American Society of Anesthesiologists Physical Status Classification System (ASA) and the number of previous revisions (Table 1).

Both groups were similar and statistically non-different regarding the indication for surgery, which was limited to aseptic loosening, wear of liner and recurrent dislocation by the case selection criteria (see flow chart Fig. 1). The indications and surgical procedures were discussed and defined in our clinic-internal indication board. All treatments are based on the evaluated standard operating procedures (SOP) of our clinic.

### Implant-related comparison

Differences in surgery- and implant-related data between the two groups were partly significant and are listed in Table 2. In the SG, the fixation technique used for most prostheses was cemented (35%), followed by hybrid (20%) and uncemented (19%). The larger part of the CG prostheses was implanted uncemented (48.5%), followed by cemented (15%).

The average number of the exchanged femoral stems was 14% in the SG and 24% in CG, whilst acetabular cups were exchanged at a rate of 25% in the SG and 12% in the CG. In summary, there were no significant differences between the two groups regarding the components exchanged during R-THA.

Mean femoral-head-size and acetabular-cup-size were similar in both groups (Table 2). The mean head diameter of the dislocated cases was even 1 mm larger in the CG than in the SG (Table 2), which should actually result in a reduced dislocation risk in the CG. A higher dislocation tendency in the CG due to different head sizes is thereby excluded. The cutting-to-suture time was approximately 15 min longer in the CG (*p* = 0.042).

### Comparison of postoperative parameters

The postoperative hospitalisation period was about 8 days longer in the CG than in the SG with 17 (range 6–83) vs. 9 (range 5–19) days, respectively (*p* < 0.001).

**Fig. 3:**
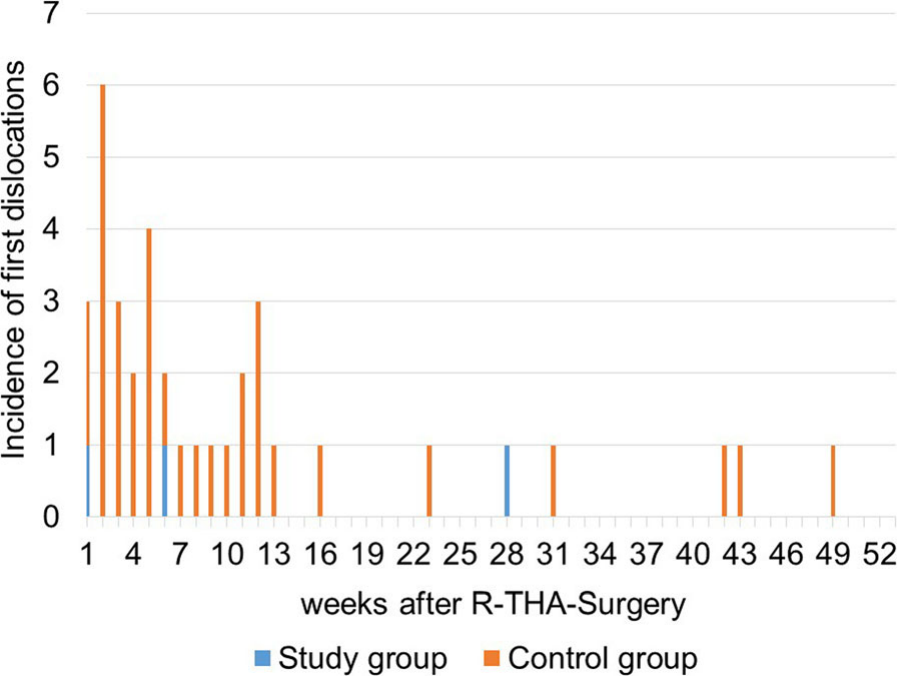
Incidence of first dislocations in a 12-months period (52 weeks) following R-THA

In the SG, the number of patients affected by dislocations was 3% (3/100) after 6 and 12 months compared to 19.5% (31/159) after 6 months and 21.5% (34/158) after 12 months in the CG, respectively (*p* < 0.001, Table 3). Because data evaluation for dislocation was restricted to the 12-months follow-up in this study, five late first-dislocation-cases (all from the CG, first dislocation occurred > 12 months after surgery) were considered “not dislocated” in order to keep the follow-up consistent for both groups. Thirty of totally 42 patients (71%) with one or more dislocations had their first dislocation incident within the first 3 months after R-THA surgery. After that period, the risk of dislocation was markedly reduced (see Fig. 3).

In the CG, 17 of 46 postoperative revisions (37%) were due to dislocation. Likewise, 17 of 39 dislocations in the CG (44%) caused surgical re-intervention within 6 months. Additionally, more than half of all patients (24/42; 57%) suffered multiple episodes of dislocations.

In terms of pain assessment, the average pain reduction postoperatively was lower in the CG with 4.41 in comparison to the SG with 5.48 (Table 4).

Moreover, all the WOMAC scores concerning pain, stiffness, physical function and global score, were better in the SG (Table 4).

### Cox regression analysis

The results of Cox regression analysis, adjusted for the overall follow-up (Table 1), are given in Table 5. Taking into account the influencing factors capsular repair vs. resection, BMI, ASA, head size and fixation technique different hazard ratios could be determined. Taking capsular resection into account as an isolated factor, we have determined a hazard ratio of 4.1 compared to capsular repair (Table 5**)**. When rating the Cox regression analysis, the small number of included dislocation events (*n* = 32) must be taken into account.

**Table 5:** Cox regression analysis of major factors influencing dislocation and with isolated consideration of the factor capsular resection vs. repair (each adjusted for the follow-up, *n* = 32 events, 12.4% of the entire sample)

	Hazard Ratio	Confidence Interval	
		Lower	Upper
Major factors			
Capsular resection vs. repair	3.8	0.9	16.5
BMI	1.0	0.9	1.0
ASA	2.5	1.1	5.3
Head size	1.2	1.0	1.3
Fixation	1.1	1.0	1.5
Isolated factor capsular treatment			
Capsular resection vs. repair	4.1	0.9	17.8

## Discussion

Application of the less-invasive modification of Bauer’s lateral transgluteal approach including capsular repair for R-THA lead to markedly reduced dislocation rates compared to the standard approach with capsular resection, indicated by 3% vs. 21% dislocation rates in a 12-months follow-up, respectively. Thus, according to these preliminary results, an estimated reduction of hip dislocations around 86% can be achieved if capsular repair is applied in combination with Bauer’s lateral transgluteal approach in R-THA in contrast to the conventional technique with capsular resection via the anterolateral Watson-Jones-approach, similar to our findings in primary THA [13].

Dislocation is considered the second most common complication of THA and the second most common reason for R-THA [2–5] after aseptic loosening. In some studies, dislocation is even the leading cause for R-THA [23]. Dislocation rates ranging from 5 to 20% after R-THA [6, 7, 9]. A population-based study reported a dislocation rate of 14.4% in 12,956 cases of R-THA [8].

**Fig. 4:**
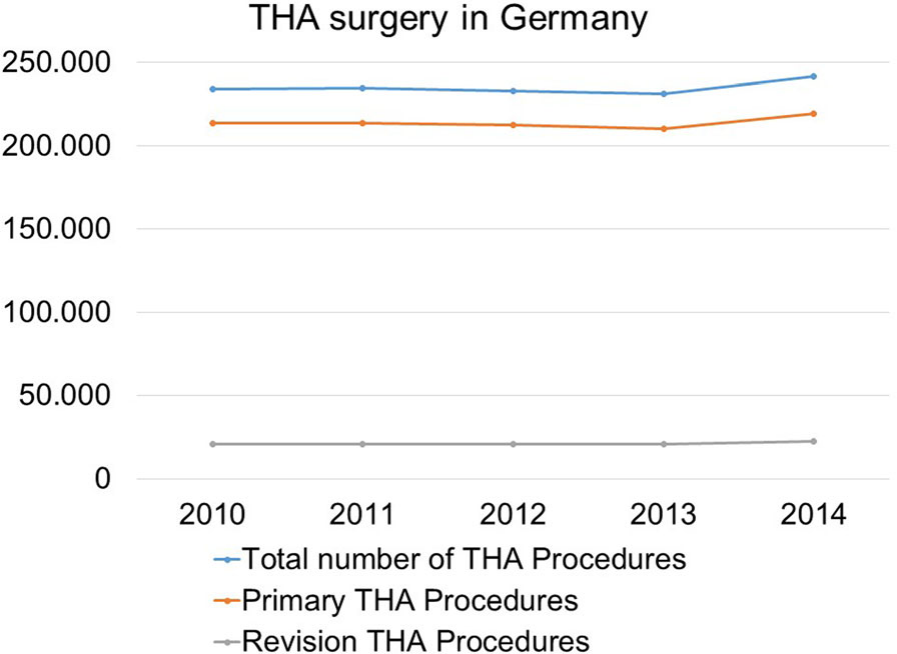
Development of Total Hip Arthroplasty-Numbers in Germany in the period between 2010 and 2014 [24]

In Germany alone, the total number of annual THA procedures was larger than 240,000 in 2014 [24]. Surgeons are therefore faced with a considerable number of THA dislocations, accounting for thousands of cases despite relatively low dislocation rates. Growing numbers can be expected because of demographic change. The total number of THA procedures performed in Germany increased from 234,349 in 2010 to 241,673 in 2014 (increase of 3.1%) [24], whereas the number of R-THA increased from 20,652 in 2010 to 22,348 in 2014 and thus from an 8.8% (2010) to a 9.2% (2014) proportion of all THA procedures (Fig. 4). These data are in line with the Swedish register, showing a rise from 1426 R-THA performed in 2002 to 2283 in 2012, which means an increment of about 60% [2, 25]. It can be assumed that the number of patients with osteoarthritis of the hip increases with higher life expectancy. This will lead to a rise in THA and R-THA procedures with their accompanying dislocation incidents. There are many patients suffering from recurrent instability and requiring further surgical intervention [26]. In addition to the medical importance of THA dislocation, the economic impact is similarly significant [27]. The combination of these factors indicates a need for the reduction of dislocation incidents.

### Risk factors for THA dislocation

The causes of THA dislocation are variable and multi factorial, including patient-related factors (age, sex, indication, co-morbidities, lack of compliance), surgery-related factors (implant misalignment, surgical approach, soft tissue handling, experience of the surgeon), and implant-related factors (head diameter, design of the liner, head-neck-ratio) [28–30]. The most important factors listed here:

- First, a correct cup position and orientation in the “safe zone” [31] or “landing zone” [32] is a prerequisite for avoiding impingement and dislocations.
- Second, the posterolateral approach is associated with a significantly higher risk of dislocation [9].
- Third, the use of larger (≥ 36 mm) or functionally larger head (bi- or tripolar hip endoprostheses) considerably decreases the dislocation rate after primary THA [15, 16, 33, 34] and R-THA [33, 34].
- Forth, there is growing evidence that preserving and reconstructing the hip joint capsule may significantly lower the dislocation rates following primary THA [13, 14, 28, 35, 36] as well as R-THA [7, 17, 18].

A combination of capsular repair and using a larger head size may restore physical joint stability on a larger scale, provided by the permanent hip-stabilizing effects of atmospheric pressure, which continuously acts on the cross-sectional area of the prosthetic head, if the joint is hermetically sealed by either the hip capsule or connective tissues in the plane of the hip joint capsule [13, 35, 37–39].

### Effects of capsular repair

The effects of capsular repair on dislocation rates after primary THA are reported in the literature. Some authors, analysing published reports on the effects of capsular repair on dislocation rates, found dislocation rates of 0% vs. 4% (*n* = 790) and 0.8% vs. 6.2% (*n* = 284) if capsular repair was performed instead of capsular resection [36]. Other studies reported about dislocation rates of 0.6% vs. 2.8% (*n* = 1000) [28] and of 0.7% vs. 4.8% (*n* = 1515) [40] if capsular repair was performed instead of capsular resection.

Other than these reports on capsular repair in primary THA, few studies can be found regarding capsular repair in combination with the posterolateral approach in R-THA. One of the aforementioned studies reported a dislocation rate of 2.5%, which was considerably lower than other reports in the literature [18]. One study including 47 R-THA cases with capsular reconstruction reported no dislocations [17]. Another group compared a capsular repair technique using 32-mm-heads (*n* = 110) with a capsular resection technique using 28-mm heads and found a reduced dislocation rate of 2.7% vs. 10.6% [7].

This study is the first to report on the decrease of dislocation rate following R-THA via Bauer’s lateral transgluteal approach. The resulting 12-months dislocation rate of 3% in the study group is relatively low and comparable to a reported rate of 2.7% using a similar capsular repair technique via posterolateral approach [7]. The 12-months dislocation rate of 21.4% in our CG appears to be relatively high compared to dislocation rates of 5 to 20% reported in the literature [25, 26]. Equally, the 6-months dislocation rates of 19.5% in the CG appeared to be higher than the 14.4%, reported in another large population-based study [8]. In our series in total 14% of all R-THA patients suffered from dislocation, which is comparable to the aforementioned studies [8, 25, 26]. Most important is the 86% reduction in dislocations after R-THA in our SG relative to the CG. The follow-up of at least 16 months and response rates of 70% underline the power of our results, since the vast majority of dislocation incidents occur in the first 6 weeks and almost all dislocations in the first 12 months after surgery [13]. Furthermore, all other complications also occur predominantly in the first 6 months after surgery [26]. These early complications accounted for more than 70% of all complications in our study.

Postoperative hospital stay time was almost halved in the SG compared to the CG. In the study period, patients were only discharged after complete removal of suture material and with non-secreting dry wounds. Due to the routinely used resorbable intracutaneous suture in the SG, there was no suture removal required. Thus, SG patients could be discharged earlier and had a shorter postoperative hospital stay time. Furthermore, there were more revisions with cup exchange (isolated cup or complete THA-exchange) in the CG. This is probably responsible for the slightly longer operation time in the CG compared to the SG with more exchanges of head and liner or stem. The difference in the ASA classification was significant and less favorable for the CG. The huge discrepancies in the dislocation rates, however, could not be plausibly explained by this nonspecific factor. The small difference in BMI between the two groups has no practical relevance (Table 1). According to the WOMAC scores, the clinical outcomes of pain, stiffness, physical function and global outcome were worse in the CG. Furthermore, patients in the CG were faced with an increased risk of surgical re-intervention, considering revision rates of 29% within 79 months in the CG and 10% within 49 months in the SG. Epidemiological- and implant-related factors of this study cannot explain the resulting differences in dislocations between the SG and CG. The results of this study underline that capsular preservation and repair is of crucial importance to avoid early dislocations and subsequent revisions after R-THA-procedures.

Some authors supported this theory by pointing out that the highest risk for dislocation occurs immediately after surgery before any pseudo-capsule has reformed in patients with previous capsular resection [38]. Beyond that, another study demonstrated that a repaired posterior capsule remained intact in 90% of patients at 3-months follow-up [19]. This is in line with the higher rate of early dislocations in the CG-series of this study (Fig. 3) as well as in the capsular resection group of our previous study, analysing primary THA cases [13]. Both results can likely be explained by the absence of a hip capsule or scar tissue within the first 3 months following the R-THA or THA procedure in both capsular resection groups before a scarred capsule is formed.

Preservation and repair of the capsule, supported by the use of larger prosthetic heads if possible, helps to re-establish more of the pre-existing protection against dislocation, which is physiologically provided by the permanent hip stabilising effect of atmospheric pressure [38]. This mechanism seems to be one of the main factors, maybe even the leading factor, in the avoidance of THA dislocation [13, 35, 37–39]. This phenomenon has been described in detail by Prietzel et al. [35, 38]. A shorter postoperative hospitalization period demonstrates that the application of the modified, less-invasive, capsule preserving R-THA technique is accompanied by further medical and economic benefits.

### Limitations

It was impossible to measure the acetabular inclination and anteversion angles in this study. However, a significant influence on the outcome is unlikely because senior surgeons with greater professional experience (Table 2) performed the R-THA-procedures in the CG.

The low number of patients included, the fact, that only one surgeon aimed at capsular repair (surgeons coincidentally assigned to the operation), the intraoperative decision-making abou**t** feasibility of capsular repair (possible selection bias), the differences in the overall follow-up periods, in the ASA classification and the number of previous revisions in both groups as well as a response rate of 70% were limitations of this study. Interpretability of Cox regression analysis was limited by the small number of dislocation events, especially in the study group with capsular repair (*n* = 3), in addition to the number of influencing factors investigated in the given study. As a further technical limitation, capsular reconstruction is impossible in about 40% of the R-THA-cases.

## Conclusion

In this retrospective analysis of R-THA-procedures we found a significant reduction of cases affected by dislocations in the 12-months follow-up (minus 86%), when the modified less-invasive and capsule repairing technique via Bauer’s lateral transgluteal approach was used instead of the conventional technique with capsular resection via the anterolateral approach. These results supplement and extend the existing literature’s findings on capsular repair in R-THA using the posterolateral approach [7, 17, 18] and on capsular repair in primary THA [13, 14, 28, 36, 40].

With a lower re-revision rate and improved WOMAC scores, there was no evidence of the often postulated but to our knowledge not demonstrated disadvantages of capsular repair such as “capsular pain” or limited range of motion. As further advantages of the less invasive technique as a combination of capsular repair and resorbable intracutaneous suture, we found a significantly shorter postoperative hospitalisation period and even a shorter cutting-to-suture time. According to our experience, the less-invasive technique can be applied to approximately 60% of all R-THA cases.

Considering these medical and economic benefits, the modified, less-invasive capsular repairing approach in R-THA may be beneficial and can therefore be recommended.
